# Auditory Sensory Gating in Children With Cochlear Implants: A P50-N100-P200 Study

**DOI:** 10.3389/fnins.2021.768427

**Published:** 2021-12-06

**Authors:** Yan-Xin Chen, Xin-Ran Xu, Shuo Huang, Rui-Rui Guan, Xiao-Yan Hou, Jia-Qiang Sun, Jing-Wu Sun, Xiao-Tao Guo

**Affiliations:** ^1^Department of Otolaryngology-Head and Neck Surgery, The First Affiliated Hospital of USTC, Division of Life Sciences and Medicine, University of Science and Technology of China, Hefei, China; ^2^CAS Key Laboratory of Brain Function and Diseases, School of Life Sciences, University of Science and Technology of China, Hefei, China

**Keywords:** auditory sensory gating, cochlear implant, P50, N100, P200, attentional dysfunction

## Abstract

**Background:** While a cochlear implant (CI) can restore access to audibility in deaf children, implanted children may still have difficulty in concentrating. Previous studies have revealed a close relationship between sensory gating and attention. However, whether CI children have deficient auditory sensory gating remains unclear.

**Methods:** To address this issue, we measured the event-related potentials (ERPs), including P50, N100, and P200, evoked by paired tone bursts (S1 and S2) in CI children and normal-hearing (NH) controls. Suppressed amplitudes for S2 compared with S1 in these three ERPs reflected sensory gating during early and later phases, respectively. A Swanson, Nolan, and Pelham IV (SNAP-IV) scale was performed to assess the attentional performance.

**Results:** Significant amplitude differences between S1 and S2 in N100 and P200 were observed in both NH and CI children, indicating the presence of sensory gating in the two groups. However, the P50 suppression was only found in NH children and not in CI children. Furthermore, the duration of deafness was significantly positively correlated with the score of inattention in CI children.

**Conclusion:** Auditory sensory gating can develop but is deficient during the early phase in CI children. Long-term auditory deprivation has a negative effect on sensory gating and attentional performance.

## Introduction

There is a close link between cognitive decline and hearing loss (Dye and Hauser, 2014; Heinrichs-Graham et al., 2021). Patients with hearing loss face the risk of delays in multiple cognitive functions, such as working memory and executive function (Lieu et al., 2020). Specifically, attention-deficit disorders are more commonly reported in deaf children compared with normal-hearing (NH) peers (Hall et al., 2018). As one of the most successful neural prostheses developed to date, cochlear implants (CIs) help not only to restore hearing of deaf children, thereby supporting speech communication, but also to enhance their cognitive abilities (Kral et al., 2019). For example, CI children showed an improvement in non-verbal cognitive functions and working memory at 6 months after CI surgery (Shin et al., 2007). However, CIs still cannot ensure optimal cognitive outcomes (Kral et al., 2019). There is a great variation in the attentional performances of CI children (Surowiecki et al., 2002). Both preschoolers and school-aged children with CIs were found to face a greater risk of deficits in the attention domain compared with NH children (Kronenberger et al., 2014). Nearly 40% of CI children attending mainstream classes could not pass the test of attention (Mukari et al., 2007), which may result in poor educational performance. However, the related neural mechanism underlying poor attentional performance in CI children remains unclear.

Previous evidence has shown that sensory gating is involved in early information processing of auditory attention (Wan et al., 2008). Sensory gating refers to the brain’s ability to filter repetitive irrelevant stimuli (Chien et al., 2019), which is mainly assessed by P50 suppression. As a “pre-attentive” process, P50 sensory gating manifests in the central nervous system modulating its sensitivity to incoming stimuli (Braff and Geyer, 1990), protecting the brain from information overload (Adler et al., 1982). The P50 is a positive component of auditory event-related potentials (ERPs) and usually occurs at about 50 ms after stimuli onset. It has been supposed to be generated from the thalamo-cortical projection to the auditory cortex (Sharma et al., 2009). In a paired-click paradigm, two successive P50 responses are usually evoked by an initial stimulus (S1) and a shortly following identical stimulus (S2). (Fruhstorfer et al., 1970). Normal P50 suppression is characterized by a reduction in P50 amplitude for S2 compared with S1. A higher ratio (S2/S1) or smaller difference in these two P50 amplitudes suggests weaker sensory gating associated with diminished cognitive functioning, such as attention (Lijffijt et al., 2009).

Given that sensory gating is regarded as a multistage process (Boutros et al., 1999), previous studies have also paid attention to the later phases of auditory processing reflected by the N100 and P200 (Rosburg, 2018). The N100 is a negative component appearing about 100 ms after the onset of the auditory stimulus, and the P200 is a positive component appearing about 200 ms. The N100 and P200 components are mainly generated in the primary auditory cortex (Hegerl and Juckel, 1993). These two components have been proposed to involve distinct neural activities (Boutros et al., 2004; Chien et al., 2019) and thus be related to different functions (Lijffijt et al., 2009). Unlike the P50 involving the early phase of information processing, the N100 and P200 are considered to reflect triggering and allocation of attention, respectively (Shen et al., 2020). Thus, different phases of auditory information filtering should be investigated by the P50-N100-P200 complex.

There is a maturational course of sensory gating in typically developing children (Davies et al., 2009). Compared with adults, children always show immature sensory gating ability as revealed by longer P50 latencies (Hunter et al., 2012). With increasing age, young children (1–8 years of age) demonstrate a rapid decrease in latency (Freedman et al., 1987). The latency may stabilize at the pre-adolescent stage (9–12 years of age) and remain stable into adulthood. Brinkman and Stauder (2007) also found a negative correlation between age and the P50 amplitude ratio, indicating age-related sensory gating abilities. However, further analysis revealed that a significant difference in gating ratios was only found between the youngest children group (5–7 years of age) and the other three groups (8–9, 10–12, and 18–30 years of age) and not among the latter three groups. These findings imply that sensory gating may mature around the age of 8 years.

Sensory gating has been reported to be deficient in many neurological diseases (Gjini et al., 2011; Micoulaud-Franchi et al., 2015). Patients with schizophrenia (Smucny et al., 2013) or autism spectrum disorders (Crasta et al., 2021) showed reduced gating abilities reflected by abnormal P50, N100, and/or P200 amplitude ratios. This ineffective inhibitory modulation of sensory information may imply an imbalance of neuronal excitation/inhibition in this population (Culotta and Penzes, 2020). The inhibitory system is thought to be the underlying mechanism in modulating sensory gating (Adler et al., 1982). Evidence has also demonstrated that peripheral auditory deafferentation or sensorineural hearing loss negatively affects inhibitory mechanisms, reflected by a reduction of inhibitory inputs and subsequent imbalance between excitatory and inhibitory systems (Campbell et al., 2020a). The properties of the inhibitory synapses in the central auditory system are changed by auditory deprivation (Takesian et al., 2012). The inhibitory activity decreases, followed by an increase in the excitability of both midbrain and cortical neurons. Synaptic changes induced by early hearing loss contribute to auditory processing deficits and may be persistent even after auditory intervention (Takesian et al., 2009). Therefore, for deaf children who experience early auditory deprivation, it is unclear whether auditory sensory gating is deficient (no or reduced inhibition of repetitive irrelevant stimuli) after cochlear implantation.

In this study, we assessed auditory sensory gating in CI children by measuring the amplitude (gating) ratios of P50, N100, and P200 responses to paired tone bursts (S1 and S2). The attentional performance was also evaluated using the Swanson, Nolan, and Pelham IV (SNAP-IV) scale. We hypothesized that the sensory gating ability could develop after cochlear implantation but still be deficient because of long-term auditory deprivation. Therefore, we predicted that P50, N100, and/or P200 suppression would be poorer in CI children than in NH peers.

## Materials and Methods

### Participants

Twenty-four native Chinese children, including 12 prelingually deafened children with unilateral Med-El CI devices [6 females, age range: 4–8 years; mean age ± standard deviation (SD): 6.01 ± 1.33 years] and 12 NH children (4 females, age range: 3.5–8.5 years; mean age ± SD: 6.59 ± 1.54 years), participated in this study. Eleven CI children did not pass the neonatal evoked otoacoustic emission test and were diagnosed with congenital sensorineural hearing loss. The other child was found to have profound sensorineural hearing loss before the age of 15 months. Two CI children had worn hearing aids before cochlear implantation. The auditory and speech abilities of CI children were evaluated by Categories of Auditory Performance (CAP), Speech Intelligibility Rate (SIR), and Meaningful Auditory Integration Scale (MAIS; Peixoto et al., 2013). The scores of these three scales and more detailed information for CI children are listed in Table 1. The NH children did not have a history of hearing loss. The two groups were matched in terms of years of education, family incomes and levels of parental education. They had normal vision and no history of neurological or psychiatric illness. The protocols and experimental procedures in this study were reviewed and approved by Anhui Provincial Hospital Ethics Committee. Each participant’s guardians had filled out an informed consent carefully before the experiment.

**TABLE 1:** Demographic information of the cochlear implant users.

Subject	Gender	Age at test (years)	CI use (years)	ABR threshold (dB nHL)		CI processor	Implant type	Age at CI (years)		MAIS	SIR	CAP
				left	right			left	right			
1	M	5.33	4.08	95	>95	Opus 1	CONCERTO	/	1.25	33	3	8
2	M	7.92	2.42	>95	>95	Opus 1	SONATA	/	5.50	31	3	6
3	M	4.00	2.00	>95	>95	Opus 2xs	SONATA	/	2.00	21	2	6
4	F	6.42	2.84	>95	>95	Opus 2xs	SONATA	3.58	/	37	5	8
5	M	4.58	2.00	>95	>95	Opus 2xs	SONATA	2.58	/	34	4	6
6	F	6.67	4.34	>95	>95	Opus 2xs	SONATA	/	2.33	38	5	7
7	M	4.83	3.83	>95	>95	Opus 2xs	SONATA	/	1.00	35	4	7
8	F	5.17	3.42	>95	>95	Opus 2xs	SONATA	/	1.75	40	5	8
9	F	7.83	6.41	>95	>95	Opus 1	SONATA	/	1.42	37	5	8
10	M	7.42	0.50	95	>95	Opus 2xs	CONCERTO	6.92	/	36	4	8
11	F	5.25	2.17	>95	>95	Opus 1	SONATA	/	3.08	34	3	6
12	F	6.75	5.25	>95	>95	Opus 2xs	SONATA	/	1.50	38	3	8

*ABR, auditory brainstem response; CAP, categories of auditory performance; CI, cochlear implant; F, female; M, male; MAIS, meaningful auditory integration scale; nHL, normal hearing level; and SIR, speech intelligibility rate.*

### Sensory Gating Paradigm

In the electroencephalography (EEG) experiment, the tone burst (1,000 Hz, 30 ms duration, 4 ms linear rise/fall time) was used as the auditory stimulus to evoke the P50, N100, and P200 components. Tone bursts were presented in pairs: a conditioning stimulus (S1) and a testing stimulus (S2) with an interstimulus interval of 500 ms and an interpair interval of 8 s through two loudspeakers placed at ± 45° azimuth, at a distance of 100 cm in front of the participants. The stimuli were delivered at an intensity of 80 dB SPL. For each participant, the experiment consisted of two blocks with 200 pairs of tone bursts in total and lasted for 30 min. The sound stimuli were generated by Adobe Audition 3.0 software (Adobe Systems Incorporated, San Jose, CA, United States) and presented by E-Prime 3.0 software (Psychological Software Tools, Pittsburgh, PA, United States).

### Attention Assessment

A Swanson, Nolan, and Pelham IV (SNAP-IV) scale was used to assess the attentional performances of NH and CI subjects. This rating scale was usually used to evaluate attentional deficits in patients with ADHD (Swanson et al., 2001). The SNAP-IV includes 26 items divided into three subscales: inattention (9 items), hyperactivity/impulsivity (9 items), and oppositional (8 items) (Swanson et al., 2001). Parents were asked to rate the items according to the daily performance of their children by selecting one of four grades (not at all, just a little, quite a bit, very much). A Higher score indicated more severe symptoms.

### Electroencephalography Recording

The EEG was recorded from a cap with 64 Ag/AgCl electrodes (SynAmps RT, Curry, United States) that were placed at the scalp according to the international 10–20 system. Another two electrodes were located at the left and right mastoids. The reference and ground electrodes were placed on the tip of the nose and the forehead, respectively. Vertical and horizontal electrooculography (EOG) signals were obtained by bipolar electrodes above and below the left eye and lateral to the outer canthi of both eyes, respectively. The EEG data were sampled at 500 Hz and filtered online between 0.05 and 100 Hz. Electrode resistances were kept under 5 kΩ. Each child was asked to watch a silent cartoon sitting on a soft couch and ignore the auditory stimuli.

### Data Analysis

Offline analysis of EEG data was conducted by EEGLAB 13.0.0b in Matlab R2013b (The Mathworks, Natick, MA, United States). Data were filtered with a bandpass setting of 10–100 Hz for the P50 component and with a bandpass setting of 4–30 Hz for the N100 and P200 components. The epochs were set at 400 ms, starting at 100 ms before the onset of the stimulus. Baseline correction was performed relative to a baseline of −100 to 0 ms. The independent component analysis was used to remove the eye movement, heartbeat, and CI artifacts from the EEG signals (Hongmei and Nan, 2017). Independent components reflecting these artifacts were identified and removed by visual inspection of the component’s properties, including the waveform, 2-D voltage map, and spectrum (Gilley et al., 2006). After artifact removal, segments containing voltage deviations exceeding ± 100 μV on any channels except for EOG channels were rejected.

The ERPs evoked by S1 and S2 were calculated by averaging individual trials. The P50 component was defined as the most positive peak between 40 and 100 ms after stimulus onset. The N100 and P200 components were determined as the most negative and positive peaks after P50 between 80 and 150 ms and between 120 and 250 ms, respectively (Crasta et al., 2021). The amplitude of P50, N100, or P200 was determined by the peak-to-peak amplitude between the peak of P50, N100, or P200 and its preceding peak with reversal polarity. The gating ratio between P50, N100, or P200 amplitude for S2 and that for S1 (S2/S1) was used to evaluate the sensory gating ability: A lower gating ratio indicated robust gating, and a higher ratio indicated attenuated gating. The electrode Cz was selected for illustration.

### Statistical Methods

One NH child and one CI child who had no robust N100 and P200 components were removed from further N100-P200 analysis. To assess whether auditory sensory gating existed in both groups, we compared the amplitude differences of P50, N100, and P200 in response to S1 with those to S2 using repeated measures analysis of variance (ANOVA) with stimulus (S1 and S2) as the within-subject factor. The differences in gating ratios, amplitudes, peak latencies, and SNAP-IV scores between two groups were further evaluated by a one-way ANOVA with group (NH and CI) as the between-subject factor. The Pearson’s correlation was performed to assess the relationship among the gating ratios, scores of inattention, and onset or duration of deafness or CI use.

## Results

### Absence of P50 Suppression in Cochlear Implant Children

**FIGURE 1:**
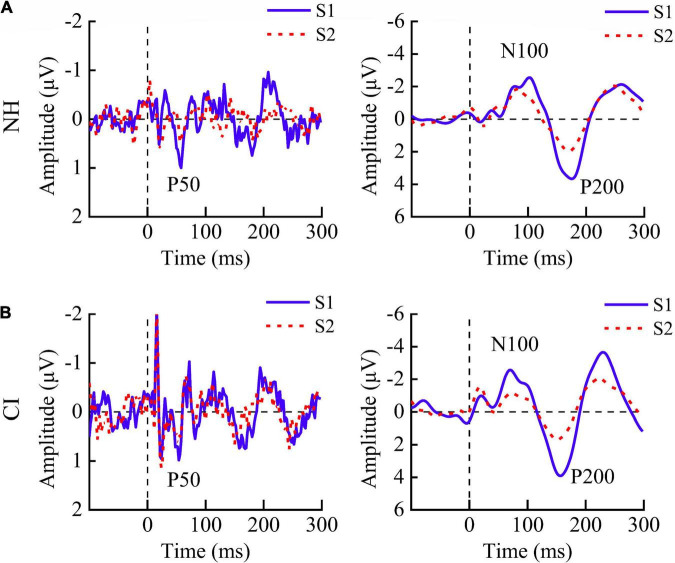
Grand average event-related potentials in response to S1 (blue solid line) and S2 (red dashed line) at site Cz. Both **(A)** children with normal hearing (NH) and **(B)** those with cochlear implants (CIs) showed robust P50, N100, and P200 components.

**FIGURE 2:**
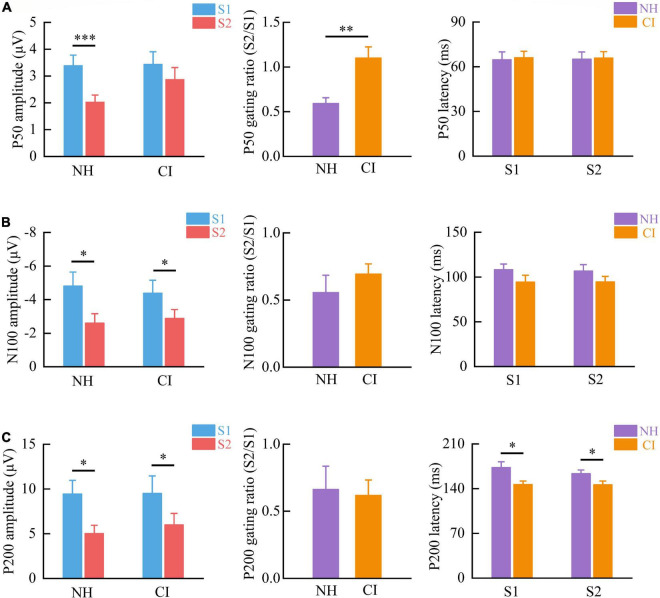
Auditory sensory gating at the **(A)** P50, **(B)** N100, and **(C)** P200 for children with NH and those with CIs. (Left) The amplitudes of N100 and P200 in response to S2 were significantly smaller than those to S1, indicating the presence of the auditory sensory gating in both NH and CI children. However, P50 suppression only existed in NH and not in CI children. (Middle) CI children showed similar N100 and P200 suppression ratios (S2/S1) but a higher P50 ratio compared with NH children. (Right) The P200 latencies in CI children were significantly shorter than those in NH children. Vertical bars represent the standard error. ^***^p < 0.001, ^**^p < 0.01, and *p < 0.05.

The grand average ERPs in response to S1 and S2 for two groups at the representative electrode Cz are shown in Figure 1. NH children showed significantly smaller amplitudes of P50 [*F*_(1,11)_ = 39.251, *p* < 0.001], N100 [*F*_(1,10)_ = 8.391, *p* = 0.016], and P200 [*F*_(1,10)_ = 9.196, *p* = 0.013] in response to S2 than those to S1, indicating the presence of robust P50, N100, and P200 suppression. CI children showed similar amplitudes of P50 [*F*_(1,11)_ = 0.348, *p* = 0.567] but significantly smaller amplitudes of N100 [*F*_(1,10)_ = 8.126, *p* = 0.017] and P200 [*F*_(1,10)_ = 8.019, *p* = 0.018] for S1 compared with S2 (Figures 2A–C, left).

### Higher P50 Ratio but Similar N100 and P200 Ratios in Cochlear Implant Children Compared With Normal Hearing Children

We further assessed whether the gating ratios, amplitudes, and peak latencies of P50, N100, and P200 differed between NH and CI children. CI children showed a significantly higher P50 gating ratio than NH children did [*F*_(1,22)_ = 13.450, *p* = 0.001] (Figure 2A, middle). However, no significant difference in N100 [*F*_(1,20)_ = 0.855, *p* = 0.366] or P200 [*F*_(1,20)_ = 0.047, *p* = 0.831] gating ratios was found between these two groups (Figures 2B,C, middle).

There was no significant difference in P50 [S1: *F*_(1,22)_ = 0.026, *p* = 0.873; S2: *F*_(1,22)_ = 3.704, *p* = 0.067], N100 [S1: *F*_(1,20)_ = 0.138, *p* = 0.714; S2: *F*_(1,20)_ = 0.131, *p* = 0.721], or P200 amplitudes [S1: *F*_(1,20)_ < 0.001, *p* = 0.979; S2: *F*_(1,20)_ = 0.367, *p* = 0.551] between NH and CI children. Peak latencies of P50 [S1: *F*_(1,22)_ = 0.037, *p* = 0.848; S2: *F*_(1,22)_ = 0.016, *p* = 0.899] and N100 [S1: *F*_(1,20)_ = 1.917, *p* = 0.181; S2: *F*_(1,20)_ = 1.657, *p* = 0.213] were similar between the two groups (Figures 2A,B, right). However, CI children showed shorter P200 peak latencies [S1: *F*_(1,20)_ = 6.155, *p* = 0.022; S2: *F*_(1,20)_ = 4.448, *p* = 0.048] than NH children did (Figure 2C, right).

### Relationships Among Gating Ratios, Attentional Performance, and Onset or Duration of Deafness or Cochlear Implant Use

**FIGURE 3:**
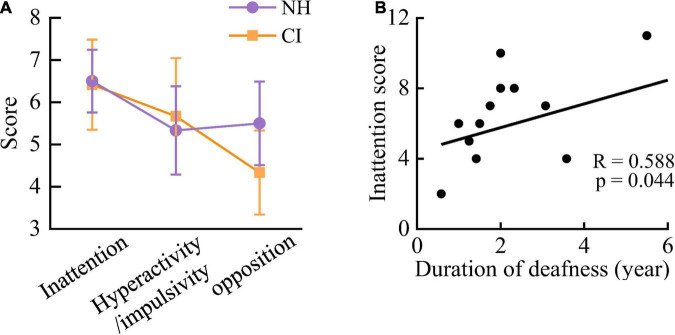
The negative effect of long-term auditory deprivation on the attentional performance. **(A)** There was no significant difference in subscale scores of inattention, hyperactivity/impulsivity and oppositional between NH and CI children. **(B)** The score of inattention was significantly positively correlated with the duration of deafness in CI children. Vertical bars represent the standard error.

No significant difference in scores of inattention [*F*_(1,22)_ = 0.004, *p* = 0.949], hyperactivity/impulsivity [*F*_(1,22)_ = 0.037, *p* = 0.849], or opposition [*F*_(1,22)_ = 0.692, *p* = 0.414] was found between NH and CI groups (Figure 3A). In CI children, the score of inattention was significantly positively correlated with the duration of deafness [*R* = 0.588, *p* = 0.044] (Figure 3B). No other significant correlations were found (all *p* > 0.05).

## Discussion

In this study, we assessed auditory sensory gating in CI children. CI children showed robust N100 and P200 suppression but no P50 suppression. Furthermore, the duration of deafness was positively correlated with the score of inattention. Our results demonstrate that auditory sensory gating can develop in CI children but is deficient during the early phase. Long-term auditory deprivation negatively affects the restoration of auditory sensory gating and attentional performance.

Cochlear implant children showed auditory gating as revealed by the N100 and P200 suppression, indicating that the CI helps to rehabilitate the auditory sensory gating abilities of deaf children. The precise organization of neuronal circuits in the mature brain is established by developmental processes that involve reorganization and fine tuning of immature synaptic networks (Kandler, 2004). The maturation of the auditory system requires stimulation. Auditory deprivation may keep the synapses immature until the cochlear implantation helps to restore hearing and get rid of this frozen state (Sharma et al., 2002a). These activity-dependent processes may include improvement in synaptic efficacy and increased myelination (Gordon et al., 2003). The auditory system may rapidly develop within a critical period of 3–6 months after cochlear implantation and enter a maturation period after 12 months (Ni et al., 2021). Most CI children in our study received implantation before 3.5 years old and still had high plasticity of the auditory cortex (Manrique et al., 1999). Therefore, sensory gating can develop and be functional in CI children, though its developmental trajectory may be delayed. Considered as an automatic and involuntary first part in the attentional processes, sensory gating may prevent limited attentional resources from being disturbed by repetitive irrelevant stimuli and protect CI children from later attentional dysfunction (Hutchison et al., 2017).

Interestingly, compared with NH children, CI children showed similar gating ratios at the N100 and P200 but no robust P50 suppression, indicating deficient sensory gating during the early phase. There are two functionally distinct generators that are related to the P50 suppression, the temporal lobe and the frontal lobe (Weisser et al., 2001; Korzyukov et al., 2007; Campbell et al., 2020b). A considerable body of invasive and non-invasive research on sensory gating suggests that the auditory P50 response may be explained by contributions from the bilateral temporal lobes, including the left and right superior temporal gyri (STG; Lee et al., 1984; Liegeois-Chauvel et al., 1994; Knott et al., 2009; Mayer et al., 2009). In addition to the bilateral temporal lobes, the prefrontal source is usually attributed to the reduction of amplitudes to repeated stimuli (Grunwald et al., 2003; Korzyukov et al., 2007). In an MEG study on M50, the neuromagnetic counterpart of the P50 component, the prefrontal region was found to suppress the activity of the bilateral STG within the auditory M50 network (Josef Golubic et al., 2014). Similar to the P50 component, the N100 and P200 gating responses involve the activation of inhibitory frontal and temporo-prefrontal networks (Campbell et al., 2020b). However, functions of strong suppression regions may be differential. The P50 gating may work as a bottom-up process, while the N100 and P200 are mainly concerned with top-down processes (Boutros et al., 2013). Incoming sensory inputs first activate automatic central inhibitory mechanisms prior to top-down cognitive involvement (Javitt and Freedman, 2015). Evidence suggests that the N100 and P200 gating may be more susceptible to attention compared with early P50 gating (Rosburg et al., 2009). For the absence of P50 suppression and the presence of robust N100 and P200 suppression in CI children, we infer that the multi-stage inhibitory networks are damaged by auditory deprivation at the early stage but can be compensated for at the later stage by top-down modulation. We also found a positive correlation between the score of inattention and the duration of deafness. These findings suggest that long-term auditory deprivation has a negative effect on both early sensory gating and attentional functions. We did not find significant correlations between the gating ratio and the attention performance. The possible reason is that the Swanson, Nolan, and Pelham IV (SNAP-IV) scale for assessment of the attentional performance depending on parents’ daily observation is relatively subjective. However, an objective and more accurate method for young children with hearing disability is indeed lacking.

Our previous study found that when dealing with complex speech sounds, CI children showed smaller and slower mismatch negativity (MMN) and even an absence of the late discriminative negativity (LDN) compared with NH children (Hu et al., 2021). Contrary to these late-latency ERPs, the robust P50-N100-P200 responses could be evoked by simple tone bursts, reflecting early processes of acoustic analysis. Compared with NH children, CI children showed similar P50 amplitudes but significantly different P50 amplitude ratios, suggesting that the brain can encode the acoustic features of novel sounds but has difficulty in inhibiting the neural response to repetitive irrelevant sounds (S2). The inhibitory system is thought to be the underlying mechanism in modulating sensory gating (Adler et al., 1982). Therefore, auditory deprivation may reduce the inhibitory activity, resulting in persistent higher excitability to repetitive irrelevant sounds during the early phase of information processing.

There are still some limitations to this study. First, while we tried to recruit CI children with consistent conditions (such as brand of CI devices), inhomogeneous aspects of CI children were still present. For example, two CI children had fitted hearing aids before cochlear implantation. We cannot separate the effect of early hearing aid fitting from that of CI use on the development of sensory gating. Therefore, a more detailed grouping method should be considered based on a larger sample size. Second, there was a lack of children implanted with the CI devices before the age of 12 months. Previous findings have shown the positive effect of early CI use on auditory rehabilitation (Sharma et al., 2002b; Dettman et al., 2007). Although we did not find correlations between the onset age of CI use and the P50-N100-P200 gating ratio, there is a possibility that earlier cochlear implantation (<12 months) may result in better rehabilitation of auditory sensory gating.

## Conclusion

The CI helps to restore auditory sensory gating in prelingually deafened children. However, this gating ability is deficient in CI children during the early phase. Long-term auditory deprivation adversely affects auditory sensory gating and attentional performance.

## Data Availability Statement

The raw data supporting the conclusions of this article will be made available by the authors, without undue reservation.

## Ethics Statement

The studies involving human participants were reviewed and approved by the Anhui Provincial Hospital Ethics Committee. Written informed consent to participate in this study was provided by the participants’ legal guardian/next of kin.

## Author Contributions

Y-XC, J-QS, J-WS, and X-TG conceived and designed the experiments. Y-XC, X-RX, X-YH, R-RG, and J-WS recruited the participants. Y-XC and X-TG performed the data acquisition. Y-XC, SH, J-WS, and X-TG analyzed the data. All authors wrote the manuscript and approved the final article.

## Conflict of Interest

The authors declare that the research was conducted in the absence of any commercial or financial relationships that could be construed as a potential conflict of interest.

## Publisher’s Note

All claims expressed in this article are solely those of the authors and do not necessarily represent those of their affiliated organizations, or those of the publisher, the editors and the reviewers. Any product that may be evaluated in this article, or claim that may be made by its manufacturer, is not guaranteed or endorsed by the publisher.
